# Staphopain mediated virulence and antibiotic resistance alteration in co-infection of *Staphylococcus aureus* and *Pseudomonas aeruginosa*: an animal model

**DOI:** 10.1186/s12896-024-00840-x

**Published:** 2024-03-04

**Authors:** Sanaz Dehbashi, Hamed Tahmasebi, Mohammad Yousef Alikhani, Mohammad-Ali Shahbazi, Mohammad Reza Arabestani

**Affiliations:** 1 Department of Laboratory Sciences, Varastegan Institute of Medical Sciences, Mashhad, Iran; 2 School of Medicine, Shahroud University of Medical Sciences, Shahroud, Iran; 3 Department of Microbiology, Faculty of Medicine, Hamadan University of Medical Sciences, Hamadan, Iran; 4 Department of Biomedical Engineering, University Medical Center Groningen, University of Groningen, Antonius Deusinglaan 1, 9713 Groningen, AV, The Netherlands; 5 Infectious disease Research center, Hamadan University of Medical Sciences, Hamadan, Iran

**Keywords:** *Staphylococcus aureus*, *Pseudomonas aeruginosa*, Co-infection, Enzymes, Enzyme therapy, Staphopain A, Cysteine protease, Recombinant

## Abstract

Polymicrobial communities lead to worsen the wound infections, due to mixed biofilms, increased antibiotic resistance, and altered virulence production. Promising approaches, including enzymes, may overcome the complicated condition of polymicrobial infections. Therefore, this study aimed to investigate Staphopain A-mediated virulence and resistance alteration in an animal model of *Staphylococcus aureus* and *Pseudomonas aeruginosa* co-infection. *S. aureus* and *P. aeruginosa* were co-cultured on the L-929 cell line and wound infection in an animal model. Then, recombinant staphopain A was purified and used to treat mono- and co-infections. Following the treatment, changes in virulence factors and resistance were investigated through phenotypic methods and RT-PCR. Staphopain A resulted in a notable reduction in the viability of *S. aureus* and *P. aeruginosa*. The biofilm formed in the wound infection in both animal model and cell culture was disrupted remarkably. Moreover, the biofilm-encoding genes, quorum sensing regulating genes, and virulence factors (hemolysin and pyocyanin) controlled by QS were down-regulated in both microorganisms. Furthermore, the resistance to vancomycin and doripenem decreased following treatment with staphopain A. According to this study, staphopain A might promote wound healing and cure co-infection. It seems to be a promising agent to combine with antibiotics to overcome hard-to-cure infections.

**Supplementary Information:**

The online version contains supplementary material available at 10.1186/s12896-024-00840-x.

## Introduction

Biofilm formation is a significant characteristic of polymicrobial communities in infections such as wounds, diabetic foot, and cystic fibrosis. Hence, decreased efficacy of chemotherapies, immune evasion, and enhanced pathogenicity provoke complicated consequences. *Staphylococcus aureus* and *Pseudomonas aeruginosa* are isolated most frequently from polymicrobial infections. Moreover, *S. aureus* and *P. aeruginosa* indicate a unique adaptation capacity, which consequences in appearance of persisting strains and complicate the infection condition. The mentioned properties are regulated by some neat and complex signaling pathways, which are controlled by Quorum sensing systems [1–3]. Beyond the partial antagonism or synergism, the relationship between these two microorganisms seems to be an overwhelming cooperation [4].

The biofilm structure provides a protecting area for the microorganisms to evade the host’s immune system and antibiotic therapy. The antibiotic efficacy reduces due to the biofilm condition, including low oxygen, decreased metabolism of microorganisms, and extracellular matrix [5]. Therefore, degradation of biofilm integrity leads to metabolism activation and increased susceptibility to antibiotics [6, 7].

Virulence production and antibiotic resistance are influenced during co-infection. Alginate overproduction by *P. aeruginosa* increases the tolerance of *S. aureus* to vancomycin [8]. The cell-free suspensions of *P. aeruginosa* result in tolerance to β-lactams, aminoglycosides, macrolides, and glycopeptides. On the contrary, *P. aeruginosa* promotes the susceptibility to fluoroquinolones and antiseptics, including chloroxylenol [9]. The same effect has been reported for *S. aureus*, which resulted in increased resistance to different antibiotics in *P. aeruginosa*. Alteration in resistance gene expression or releasing some metabolites are the most common reasons for changes in antibiotic susceptibility.

Antibiotic susceptibility is not the sole target of cooperation during polymicrobial infections. Significant alterations also occur in virulence and metabolism. Virulence factors, including toxins and degrading enzymes, are downregulated due to biofilm formation. The quorum sensing (QS) systems, including LasI/R, RhlI/R, PQS (in *P. aeruginosa*), RNAII, and RNAIII (in *S. aureus*) are up-regulated to synchronize the process among microorganisms involved in the biofilm. Some studies reported various combinations, including quaternary ammonium compounds, curcumin, chlorquinaldol, 2(5 H)-furanone, and enzymatic components, to improve the effect of antimicrobials on mixed biofilms [10].

Staphopain (*scpA* encoding enzyme) is a papain-like cysteine protease secreted by *S. aureus*. It lyses proteins in the host’s connective tissue, including fibrinogen, elastin, fibronectin, and kininogen [11]. Moreover, staphopain B degrades LL-37, an immune peptide in the skin. Therefore, the degraded peptide fails to inhibit biofilm formation [12]. In addition to the host proteolytic activity of staphopain, it exerts the capability to degrade the biofilm structure in *S. aureus* [13].

The current study aimed to investigate the effect of staphopain A on the dispersal of the dual-species biofilm in a wound infection in an animal model. Moreover, degradation of biofilm structures and microorganism release to the planktonic form might influence the QS-mediated virulence factor production. Therefore, the staphopain A mediated alteration to virulence were investigated.

## Methods

### Strains and growth condition

*S. aureus* and *P. aeruginosa* strains used in this study (obtained from microbial bank of Hamadan University of Medical Sciences) are listed in Table 1. *S. aureus* strain was tested to not produce staphopain A using casein digestion spectrophotometric method (Supplementary file 1). The ethics committee of Hamadan University of Medical Sciences approved this study and was conducted under the ethical approval code IR.UMSHA.REC.1396.694.

**Table 1:** Strains used in this study

Strains used for mono- and co-infectionss				
Strains	Species	Source	Characteristics	
			Virulence	Antibiotic Susceptibility
PA-1	*P. aeruginosa*	Wound	Toxin-producing strain/ Pyoverdine Producer	MDR strain
SA-1	*S. aureus*	Wound	PVL (Panton-Valentine Leukocidin) producing/ biofilm-forming strain/ Siderophore producer/ Non- staphopainA producer	MDR strain
	*Staphylococcus aureus* ATCC25923		control strains	
	*Pseudomonas aeruginosa* PAO1		control strains	
**Strains used for production of recombinant Staphopain A**				
Strains			Characteristic	
*E.coli* TOP10			Host for cloning DNA vectors	
*E.coli* BL21			Host for expression recombinant vectors	
*p*ET26b			His tagged C-terminal, Kanamycin resistant, *PelB* sequence	

Mannitol salt agar (MSA) (Merck, Germany) and Columbia agar (Merck, Germany) containing 5% sheep blood were used for isolation of *S. aureus*. Also, *P. aeruginosa* was cultivated using cetrimide agar (CA) (Merck, Germany). The plates were incubated at 37 °C and ambient air unless it is mentioned in the text.

### Recombinant staphopain a production

Recombinant Staphopain A was produced using the cloning method in *p*ET26b vector and *E. coli* BL21 as an expression host. Primers for cloning were designed based on the sequence of the *scpA* gene in the Gene Bank. Two restriction enzymes, *BamHI* and *XhoI* (Thermofisher, USA), were used to provide sticky ends in vector and insert sequences. The double-digested sequences were ligated using T_4_ ligase (Thermofisher, USA). Then, the ligated vector was transformed to the competent *E. coli*Top10. The recombinant vector was proved using colony PCR and sequencing the extracted recombinant vector.

The isolated recombinant vector was transformed to the expression host (*E. coli* BL21), and the protein was expressed in exposure to Isopropyl ß-D-1-thiogalactopyranoside (IPTG). The recombinant protein was confirmed using sodium dodecyl sulfate–polyacrylamide gel electrophoresis (SDS-PAGE) and Anti His-tagged western blotting. Finally, staphopain A was purified using immobilized metal affinity chromatography by cobalt resin (Talon, Takara). The protein purity was measured by High-Performance Liquid Chromatography (HPLC), and the functionality of the recombinant enzyme was determined using the casein digestion spectrophotometric method. The detailed description of cloning, expression, protein purification, and enzyme confirmation is present in supplementary file (Supplementary file 1).

### Cell culture

The mouse fibroblast cell line (subcutaneous connective tissue) - L929 acquired from the Pasteur Institute of Iran was used to investigate the staphopain effect on the co-culture of *S. aureus* and *P. aeruginosa*. The cell line was cultured as described in the study of Tahmasebi et al. [4]. Briefly, 10% FBS (Invitrogen, USA) and 50–100 IU/mL penicillin-streptomycin (Sigma, USA) were added to the DMEM medium (DNA BioTech, Iran) to culture the cell line in 24-wells plates for other investigations.

### Cytotoxicity test

MTT test (DNA BioTech, Iran) was carried out to determine the cytotoxicity of staphopain A. L929 cells were sub-cultured in a 96-well plate at a density of 5 × 10^3^ cells/well, and incubated at 37 °C and 5% CO_2_. Then, cells were treated using different concentrations of staphopain A for 16 h, incubated as previously. Following the incubation, cells were washed by PBS, fresh media and MTT solution were added. After 4 h of incubation, dimethyl sulfoxide was added to each well. Then, the absorbance was measured at 570 nm using a microplate reader (Bio-Rad, USA) Supplementary file 2) [14]..

### Co-culture on fibroblast cell line

As the monolayer reached 90% confluency, the DMEM medium was removed, and 100µL of bacterial suspensions (at exponential phase) with the OD_600_: 0.1 were added to each well containing 1mL MEM supplemented with L-Glutamine. The plates were incubated at 37 °C and 5% CO_2_ for 24 h. At 1, 6, 12, and 24 h after incubation, the media were aspirated, and fresh MEM media containing L-Glutamine were added to each well. The aspirated media were diluted in PBS and plated on MSA and CA to recover *P. aeruginosa* and *S. aureus*. After 24 h, the media were aspirated and the plates were washed with PBS twice. Then, each well was treated with 200 µl of 0.1% Triton X-100, the plate gently agitated for 30 min. Next, the cells were scraped to disrupt the biofilm. The bacteria were diluted and plated as described for the planktonic co-culture [15]. Each experiment was done in triplicate.

The activity of staphopain A was assessed on the L-929 cell line. As described previously, after the biofilm was established on the cell line, 16µL/mL of staphopain A was added to each wells of co- and mono-cultures. The media were replaced with fresh media containing staphopain A every 2 h. Then, the colonies were counted as mentioned above.

The combination of staphopain A/vancomycin (16µL/mL: 16µL/mL) and staphopain A/doripenem (16µL/mL:32µL/mL were investigated on the L-929 cell line as described for staphopain A.

### Animal model and ethical issues

All experiments were done according to the guidelines for maintenance, surveillance and usage of laboratory animals published by the National Institute of Health United State (NIH publication No. 85 − 23, revised 1985). Moreover, the study was approved by the ethics committee of the Hamadan University of Medical Sciences (No: IRUMSHA. REC. 1396.694).

The male, pathogen-free Balb/C mice which purchased from laboratory animal center of Hamadan university of medical sciences, were used in this study. Four groups (5 mice in each group) of 25 ± 2 gr male mice aged 6–8 weeks were selected, as the minimum sample size for animal models, according to Grada and et al. [16]. Groups were divided to mono-infection groups (*S. aureus* and *P. aeruginosa* separately) and co-infection group (infected with both species). Then, each groups were categorized as control and treated ones (mono- and co-infection). Twelve hours of light/dark cycle and free access to chows and water were maintained for animals, and they were kept in sterile cages individually. All the experiments on animal model were performed based on the international Ethical Conduct in the Care and Use of Nonhuman Animals in Research.

### Wound infection in an animal model and staphopain treatment

The Staphopain A activity was evaluated using a Balb/C mouse model of wound infection. To establish a wound model in an animal, a full thickness excisional wound of 1 cm diameter was created using a sterile punch. Then, the wound was infected with 50 µL of 10^5^ CFU/mL suspension of *S. aureus* and/or *P. aeruginosa*. Afterward, the wound was covered aseptically with semi-permeable polyurethane film (Hydrofilm, Hartmann, GB). The mice were monitored and the dressing was changed daily.

Staphopain A was directly applied to the wound twice a day in a volume of 25 µL (200 ng/mL). Swab samples were collected from the wound bed daily and after treatment. Moreover, the dressings were transferred into sterile PBS and plated on species-specific media to count the viable colonies. Then, antibiotic resistance and virulence factors production were determined as mentioned previously [17].

### Total RNA isolation and RT-PCR

RNA isolation was carried out on swab and the dressing samples on days 0, 5, and 10 after treatment. RNA isolation and cDNA synthesis were performed by GeneAll cDNA synthesis kit (GeneAll, South Korea) based on manufacturer’s instruction (GeneAll, South Korea). The quality of RNA and cDNA was measured using gel electrophoresis and spectrophotometric method (Thermofisher, USA).

The expression levels of *lasI/R*, *rhlI/R*, *rpoN*, *kpc*, *RNAII*, *RNAIII*, *sigB*, *walK/R* were investigated using primers listed in Table 2. *gmk* and *rpoD* were used as reference genes [13]. The reactions of a final volume of 20 µl were prepared using 2X Syber Green PCR Master Mix (Amplicon, Denmark), primers (20 pmol), and cDNA, and DEPC-treated water. The following program was applied for amplification: 95 °C for 15 min, 40 cycles of 95 °C for 20 s, and 56 °C for 30 s. All the tests were performed in triplicate and three days.

**Table 2:** Primers list

Gene	Function	Primer sequence	Reference
*RNAIII*	*S. aureus* QS gene	F: GCACTGAGTCCAAGGAAACTAACTCT R: AGCCATCCCAACTTAATAACCATGT	1
*rhlI*	*P. aeruginosa* QS gene	F: TTCATCCTCCTTTAGTCTTCCC′ R: TTCCAGCGATTCAGAGAGC	2
*rhlR*	*P. aeruginosa* QS gene	F: TGCATTTTATCGATCAGGGC R: CACTTCCTTTTCCAGGACG	2
*lasI*	*P. aeruginosa* QS gene	F: TTTGGATCCTATACTCTCTGA R: ACGCAACTTGTGGATCCCGC	12
*lasR*	*P. aeruginosa* QS gene	F: AAGTGGAAAATTGGAGTGGAG R: GTAGTTGCCGACGACGATGAAG	2
*algD*	*P. aeruginosa* virulence gene (alginate production)	F: ATGCGAATCAGCATCTTTGGT R: CTACCAGCAGATGCCCTCGGC	12
*rpoD P. aeruginosa*	*P. aeruginosa* housekeeping gene	F: GGGCTGTCTCGAATACGTTGA R: ACCTGCCGGAGGATATTTCC	11
*nucA S. aureus*	*S. aureus* reference gene	F: AGCCAAGCCTTGACGAACTAAAGC R: GCGATTGATGGTGAT ACGGTT	28

### Statistical analysis

One-way ANOVA, two-way ANOVA, and Student’s t-test were performed for all the collected data by GraphPad Prism 6.0 (Graph Pad Software, USA). The Tukey and Holm-sidak tests were done as multiple comparison tests where was appropriate, based on a p-value of 0.05, as significant. All data were shown as mean ± SEM.

## Results

### Dual effect of Staphopain A on the wound infection

**Fig. 1:**
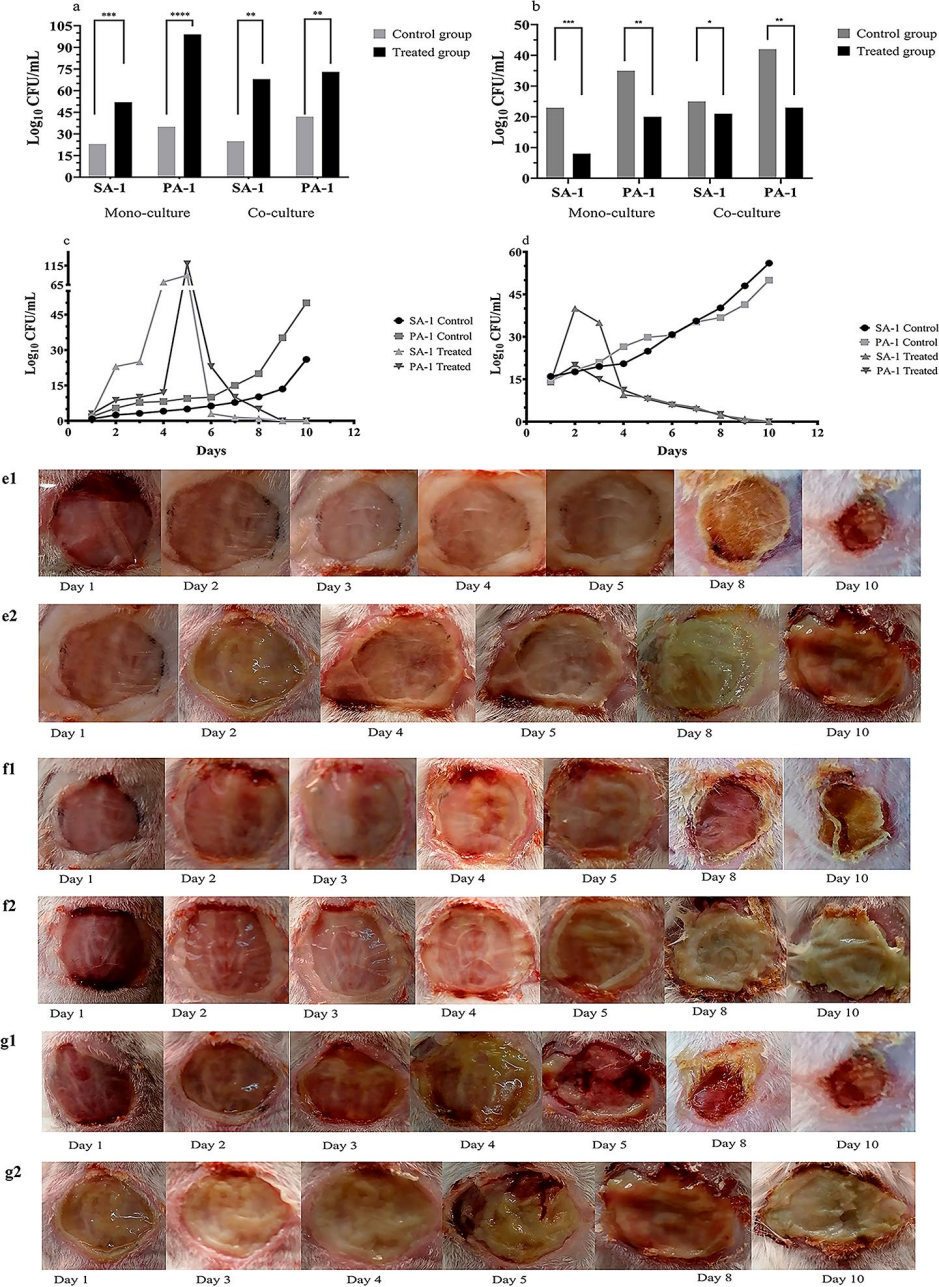
Investigation of the effect of staphopain A on viability and biofilm disruption in cell line and animal model. **a**: The viable colony counts of *S. aureus* and *P.aeruginosa* on L-929 cell line in treatment and control groups. The recovered colonies from co- and mono-cultures were counted in the planktonic condition. **b**: The viable colony counts of *S. aureus* and *P.aeruginosa* on L-929 cell line in treatment and control groups. The recovered colonies from co- and mono-cultures were counted in the biofilm condition. **c**: The viable colonies recovered from the wound co-infected with *S. aureus* and *P. aeruginosa* during 10 days of treatment with staphopain A. It seems that the biofilm was disrupted during 5 days of treatment and the viable colonies were increased, then the killing effect of staphopain A led to a decrease in colony counts. **d**: The viable colonies recovered from the wound infected with *S. aureus* and *P. aeruginosa* (mono-infection group) during 10 days of treatment with staphopain A *S. aureus* and *P. aeruginosa* strains used in this study weakly formed biofilm and the higher initial counts seem to be due to weak single biofilms. **e**: The wound healing process during 10 days of experiment in co-infections groups of control and treatment. Panel e1 indicated the co-infection group during days 1 to 10 after treatment. As depicted, the wound drainage decreased, the wound diameter reduced significantly, and the infection healed successfully. While in the control group (row e2) the wound infection worsened during 10 days. **f**: The wound healing process during 10 days of experiment in groups infected with *S. aureus*. The wound healing process in the treatment group (row f1) improved successfully during 10 days in comparison to the control group (row f2). **g**: The wound healing process during 10 days of experiment in groups infected with *P. aeruginosa*. The wound healing process in the treatment group (row g1) improved successfully during 10 days in comparison to the control group (row g2). Each data set was analyzed using the two-way ANOVA, and the Holm-Sidak method for multiple comparisons. The data were presented as Mean + SEM. * p-value < 0.05; ** p-value < 0.01; *** p-value < 0.001; **** p-value < 0.0001

As depicted in Fig. 1, a dual-impact of staphopain A was observed, (1) disruption of the mixed biofilm formed in the wound, and (2) decrease the viability of *S. aureus* and *P. aeruginosa*.

The biofilm disrupting property of Staphopain A was observed in co-culture experiments on the L929 cell line and an in vitro model of biofilm using the crystal violet method. The colony counts of *S. aureus* and *P. aeruginosa* reduced significantly in the biofilm phase in comparison to the planktonic one (Fig. 1a and b) Supplementary file 2).

Based on the data demonstrated in Fig. 1c, the colonies released to the planktonic phase have increased during five days post-treatment compared to the co-infection control group. According to Fig. 1d, a remarkable reduction in viability was detected in the mono-infection of *S. aureus* and *P. aeruginosa*. A steady decline in colony counts was noticed for mono-infections of *S. aureus* and *P. aeruginosa* in treated groups. Although biofilm formations in mono-infection groups were not detected as strong as co-infection ones (higher initial colony counts), the weak, established biofilm was damaged in these groups (Fig. 1d). Also, a significant decrease in viable colonies was observed in co-infection groups during days 5 to 10 (Fig. 1c), in which the wound closure process improved, and finally, any isolates of *S. aureus* and *P. aeruginosa* were not recovered. In control groups (mono- and co-infection), a substantial increase in colony counts, purulent drainages, and spread to healthy adjacent tissue were discovered (Fig. 1e and g).

### Staphopain a altered the expression levels of genes involved in QS and reduced the biofilm formation

**Fig. 2:**
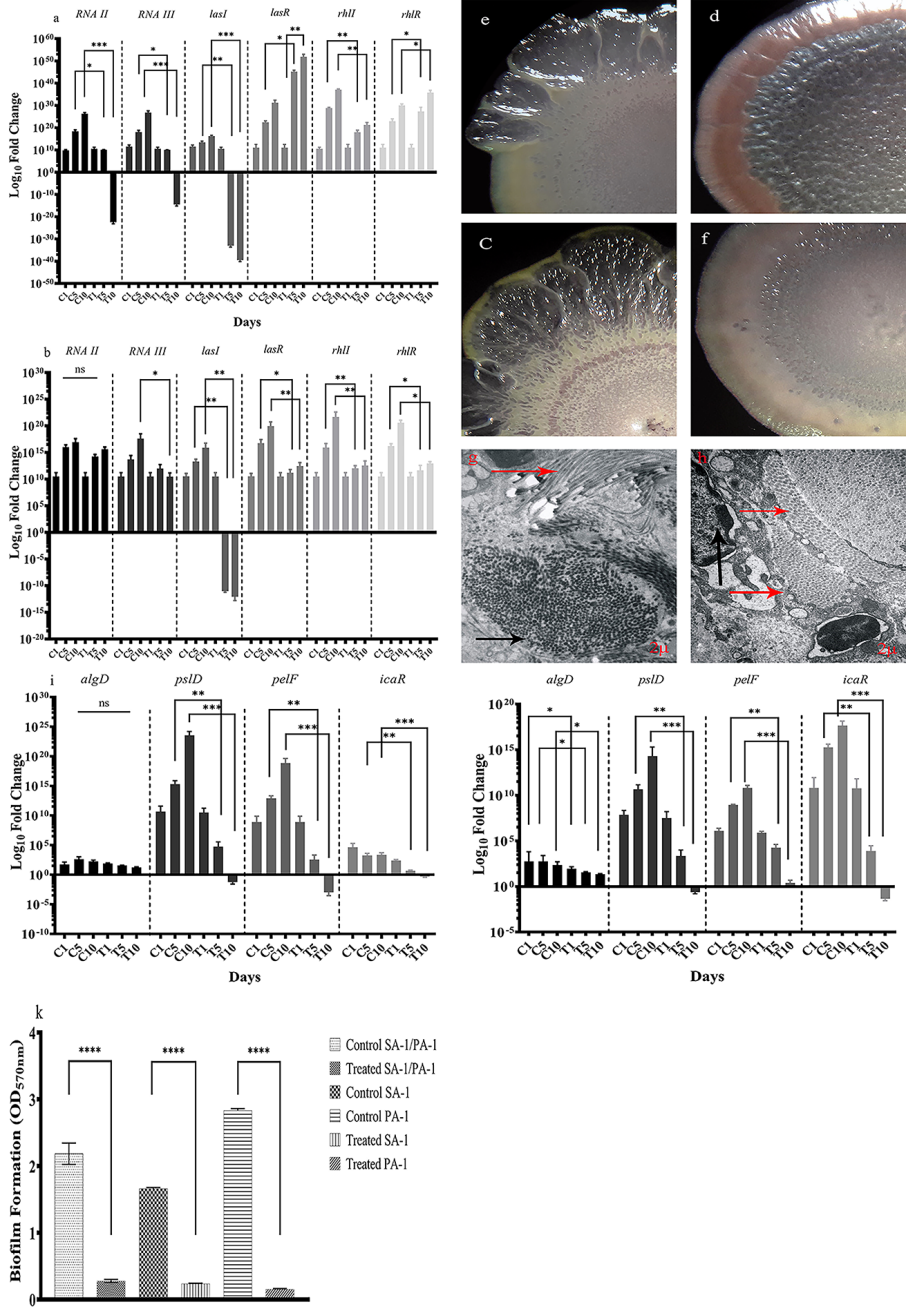
The bacterial gene expression levels in treated and control groups in animal models. **a**: The expression level of *RNAII, RNAIII* of *S. aureus* and *lasI/R*, *rhlI/R* of *P.aeruginosa* co-infection groups. C_1_, C_5_, and C_10_ indicate the control groups on days 1, 5, and 10 after infection. T_1_, T_5_, and T_10_ indicate the treated groups in days 1, 5, and 10 after infection. **b**: The expression level of *RNAII, RNAIII* of *S. aureus* and *lasI/R*, *rhlI/R* of *P.aeruginosa* mono-infection groups. C_1_, C_5_, and C_10_ indicate the control groups in days 1, 5, and 10 after infection. T_1_, T_5_, and T_10_ indicate the treated groups on days 1, 5, and 10 after infection. **c**: The stereomicroscope images of *P. aeruginosa* isolates recovered from the treated group on co-infection. The rough edge of the colony indicated the up-regulation of Rhl QS system. **d**: The stereomicroscope images of *P. aeruginosa* isolates recovered from the control group on co-infection. The smooth edge of the colony indicated the down-regulation of Rhl QS system. **e** and **f**: The stereomicroscope images of *P. aeruginosa* isolates recovered from treated (c) and control (d) groups on mono-infection. An effect similar to co-infection was observed in the mono-infection group, as well. **g** and **h**: The TEM images of animal tissue co-infected with *S. aureus* and *P. aeruginosa* in treatment (g) and control (h) groups. The bacteria embedded in the biofilm structure were indicated by red arrows. The magnification of the images was 2 μm. **i**: The expression level of genes regulated biofilm formation in *S. aureus* and *P. aeruginosa* in co-infection groups (treatment and control). C_1_, C_5_, and C_10_ indicate the control groups in days 1, 5, and 10 after infection. T_1_, T_5_, and T_10_ indicate the treated groups on days 1, 5, and 10 after infection. **j**: The expression level of genes regulated biofilm formation in *S. aureus* and *P. aeruginosa* in mono-infection groups (treatment and control). C_1_, C_5_, and C_10_ indicate the control groups on days 1, 5, and 10 after infection. T_1_, T_5_, and T_10_ indicate the treated groups in days 1, 5, and 10 after infection. **k**: The biofilm formation capacity of *S. aureus* and *P. aeruginosa* in co- and mono-culture experiments on L-929 cell line in control and treatment groups. Each data set was analyzed using the two-way ANOVA, and the Holm-Sidak method for multiple comparisons. The data were presented as Mean + SEM. * p-value < 0.05; ** p-value < 0.01; *** p-value < 0.001; **** p-value < 0.0001

According to Fig. 2a and b, the expression level of RNA II– a regulator for *agr* locus- indicated a remarkable decrease in the co-infection group during days 1, 5, and 10 after treatment as compared to the control group. Moreover, RNA III was gradually reduced in the treated group compared to the control one, which was more remarkable on day 10th. In contrast to co-infection, the expression level of RNA II and RNA III increased in the mono-infection group after treatment with staphopain on days 5 and 10; however, the expression level was significantly low compared to the control group.

As depicted in Fig. 2a and b, the expression level of *lasI* and *rhlI* dramatically reduced on days 5 and 10 after treatment in the co-infection group in comparison to the control one. However, *lasR* and *rhlR* are up-regulated to respond to the low expression of autoinducers. Moreover, *feoB*, as a regulator of biofilm formation and iron homeostasis in *P. aeruginosa*, downregulated in days 5 and 10 after treatment in dual-species biofilm compared to the control group.

Contrary to co-infection, *lasI/R* and *rhlI/R* downregulated in the treated mono-infection group to approximately 5-fold lower than the control group. Consensus with gene expression levels, the stereomicroscope images indicated the changes in the appearance of colonies after treatment with staphopain A compared to the control group. The treated groups showed a rough appearance, while in the control groups, smooth colonies were observed (Fig. 2c-f).

Moreover, the disruption of mixed biofilm was observed during the treatment in comparison to the control group. The TEM microscope images from the infected tissue of the mouse demonstrated biofilm disruption in the staphopain A treated group versus control groups (Fig. 2g and h).

The authors investigated genes involved in biofilm formation to determine the effect of staphopain A on biofilm formation. Staphopain A led to repressing the biofilm encoding genes in both *S. aureus* and *P. aeruginosa*. According to Fig. 2i and j, *ica* locus in *S. aureus* and *algD*, *pslD* and *pelF* in *P. aeruginosa* downregulated remarkably in treated co-infection groups. The same repression to a lower extent was observed in mono-infection groups. Moreover, the biofilm formation reduced significantly after treatment with Staphopain A. As depicted in Fig. 2k, the optical density (570_nm_) of biofilm in the treated mono- and co-culture groups decreased in comparison with control groups.

### Phenotypic plasticity during the co-infection

**Fig. 3:**
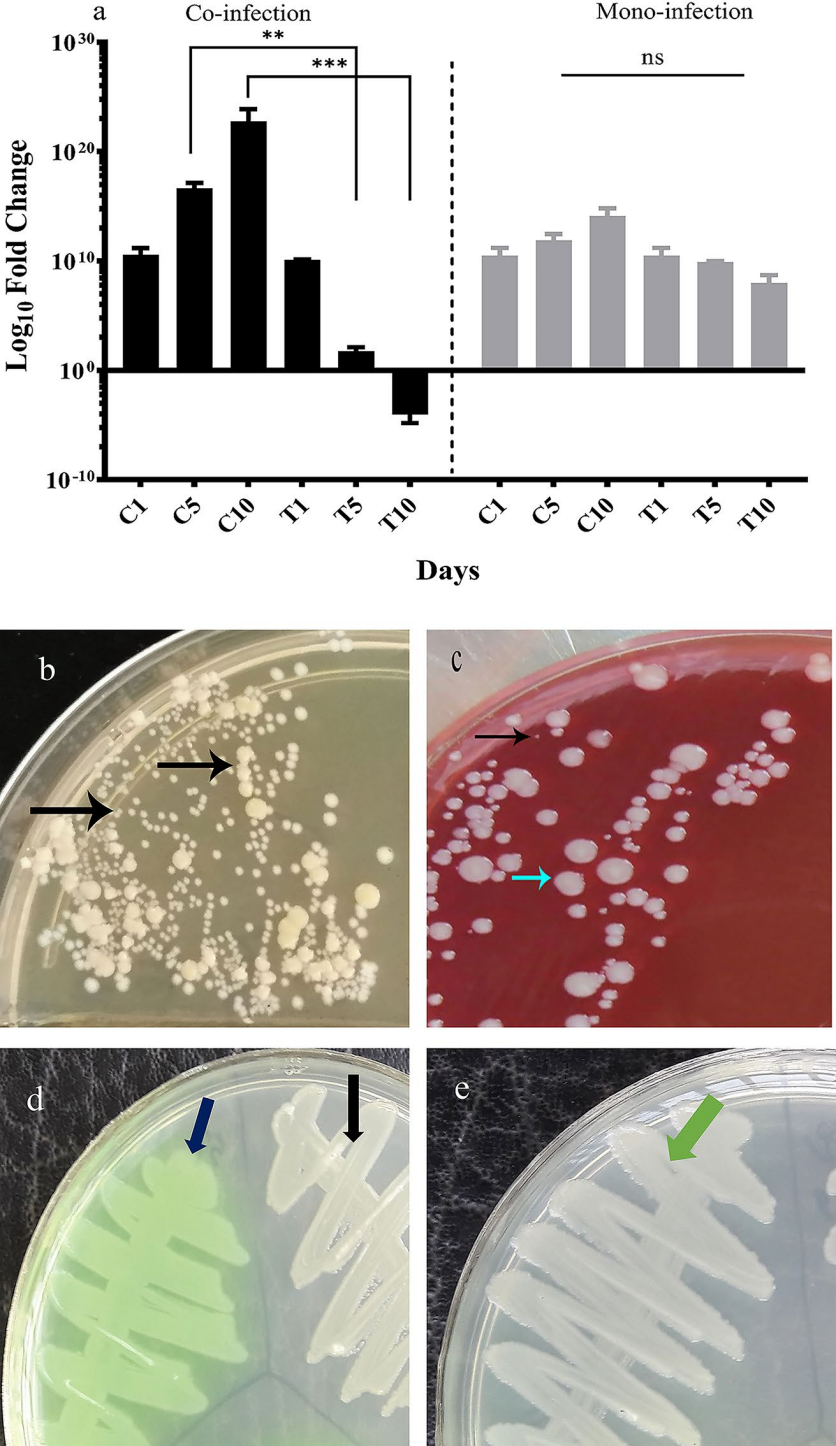
The *sigB* expression levels in treated and control groups in animal models. **a**: The expression level of *sigB* in *S. aureus* in co- and mono-infection groups. C_1_, C_5_, and C_10_ indicate the control groups in days 1, 5, and 10 after infection. T_1_, T_5_, and T_10_ indicate the treated groups in days 1, 5, and 10 after infection. **b and c**: *S. aureus* isolates were recovered in BHI agar (b) and Columbia agar supplemented with sheep blood (c). A heterogeneous population of the slow-growing, tiny isolates, and normal isolates were indicated on the plates. **d** and **e**: The pigment inactivation in *P. aeruginosa* isolates. The dark blue arrow shows pigment production in the treated co-infection group. The black and green arrows show colonies without pigment in co- and mono-infection control groups. Each data set was analyzed using the two-way ANOVA, and the Holm-Sidak method for multiple comparisons. The data were presented as Mean + SEM. * p-value < 0.05; ** p-value < 0.01; *** p-value < 0.001; **** p-value < 0.0001

According to Fig. 3a, the expression level of *sigB* significantly decreased following treatment with staphopain A in the co-infection group versus control. Contrary to the treated group, *sigB* up-regulated steadily from day1 to day 10 in the control group, simultaneous with intensifying infection and persistence of *S. aureus* isolates. As *sigB* down-regulated in the treated group, the recovered isolates from the wound normally grew on MSA. While, the recovered isolates in the control group slowly grew on MSA and indicated average growth on BHI agar supplemented with 6% NaCl and Columbia agar containing 5% sheep blood, incubating in 5% CO_2_ (Fig. 3b and c). Moreover, the same effect was observed in the mono-infection group to a lesser extent. The expression level of *sigB* was regularly reduced in the treated group in comparison to the control.

During the co-culture in the L929 cell line, *P. aeruginosa* isolates deprived the ability for phenazine production. Interestingly, the pigment was produced again after treatment with staphopain A (Fig. 3d and e). In addition, slow-growing colonies of *S. aureus* were detected after co-culture on the L929 cell line and in the co-infection model in mice. As the wound and cell line were treated with staphopain A, the morphology of *S. aureus* colonies returned to the standard type (Fig. 3b and c).

### QS-mediated virulence changed due to staphopain a treatment

**Fig. 4:**
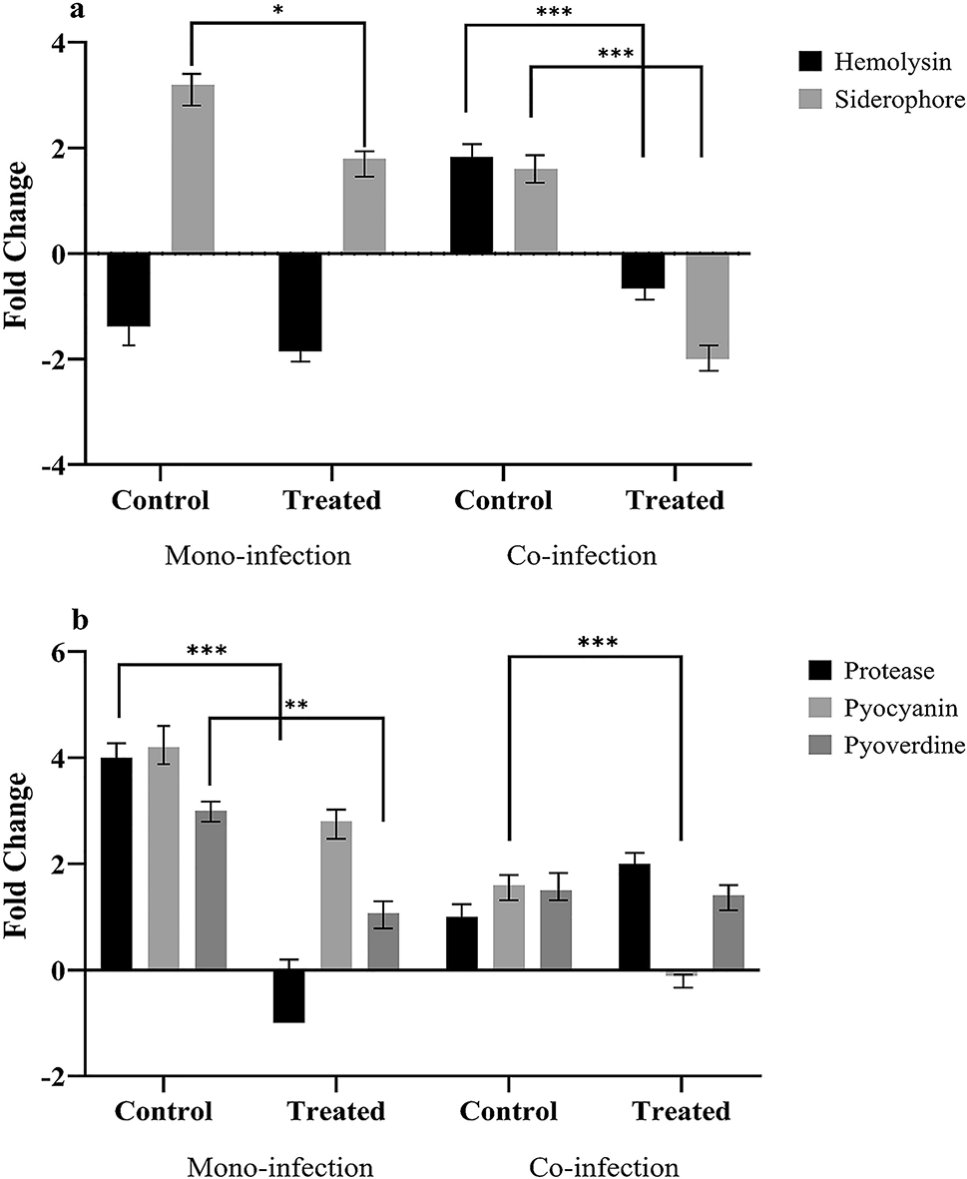
The virulence factor production in *S. aureus* and *P. aeruginosa* isolates after treatment with staphopain A. **a**: Hemolysin and siderophore production in *S. aureus* isolates on co- and mono-infection groups. **b**: Protease, Pyocyanin, and pyoverdine production in *P. aeruginosa* isolate on co- and mono-infection groups. Each data set was analyzed using the one-way ANOVA, and the Holm-Sidak method for multiple comparisons. The data were presented as Mean + SEM. * p-value < 0.05; ** p-value < 0.01; *** p-value < 0.001; **** p-value < 0.0001

As depicted in Fig. 4a, the hemolysis zone around *S. aureus* isolates decreased in the treated group compared to the control. However, hemolysin inactivation was observed in co-infection control groups, which might occur due to the persistence of *S. aureus* strains in exposure to *P. aeuginosa*.

QS-mediated virulence factors of *P. aeruginosa*, including proteases, pyocyanin and pyoverdine, were investigated. Based on Fig. 4b, protease production slightly increased in the treated co-infection group compared to the control. In contrast, a reverse effect was detected in the treated mono-infection group. The increased production of proteases (LasB and LasA) in the co-infection group synergistically raised the mortality of *S. aureus*. Staphopain A led to the down-regulation of pyocyanin in both co-infection and mono-infection groups.

Although pyoverdine production reduced remarkably in the mono-infection group after treatment with staphopain A, a slight, non-significant decline was observed in the co-infection group following treatment. Regarding the increase of LasA production and mortality in *S. aureus*, iron release to the environment caused the stability of pyoverdine in co-infection.

### Staphopain a treatment altered acquired vancomycin and carbapenem resistance

**Fig. 5:**
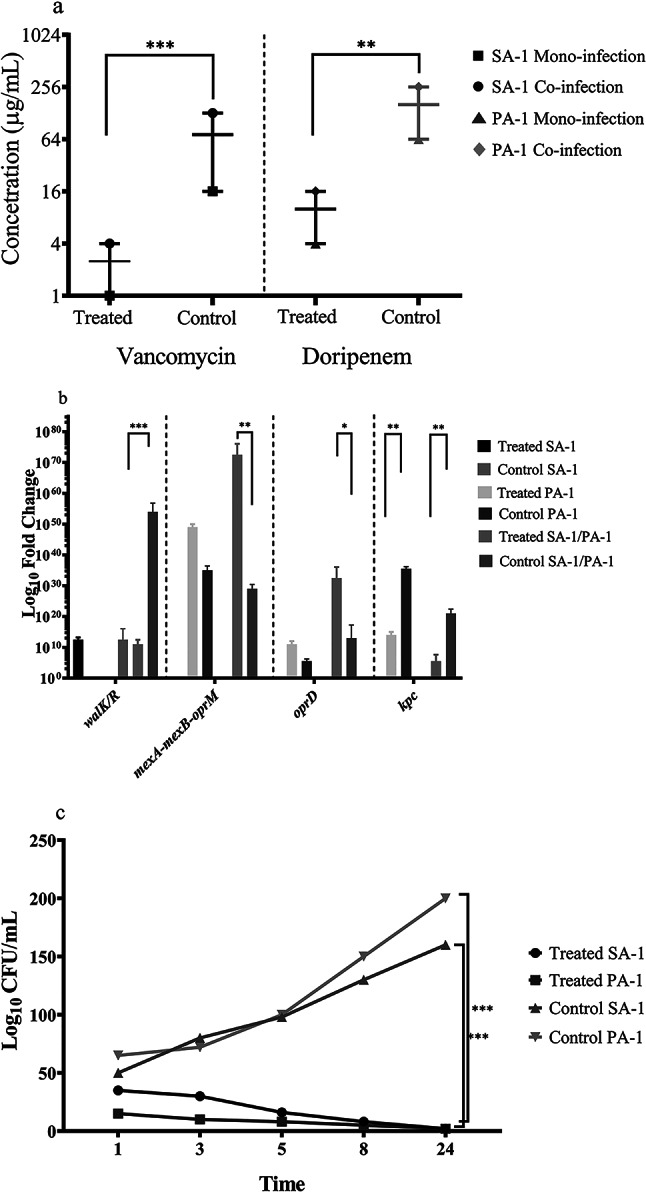
Staphopain A treatment influence on antibiotic resistance. **a**: The combination of vancomycin and Staphopain A, doripenem, and Staphopain A in co-culture group. The MIC of vancomycin and doripenem is shown in combination with Staphopain A. **b**: The expression level of *kpc*, *oprD*, and *mexA-mexB-oprM* in co- and mono-infection groups after treatment with Staphopain A. **c**: The viable colony counts of *S. aureus* and *P.aeruginosa* isolates after combination therapy in the co-culture model. The data were presented as Mean + SEM. * p-value < 0.05; ** p-value < 0.01; *** p-value < 0.001; **** p-value < 0.0001

Acquired vancomycin resistance has been previously reported in *S. aureus* and *P. aeruginosa* co-infection. Resistance to vancomycin was detected in the co-culture of *S. aureus* and *P. aeruginosa* on the L-929 cell line and animal model. Following treatment with staphopain A, the efficacy of vancomycin for the eradication of *S. aureus* increased. In the co-culture model, the combination of staphopain A and vancomycin indicated a remarkable synergistic effect. Moreover, the expression level of *walk/R* diminished, survival of *S. aureus* was reduced in both mono-culture and co-culture groups versus controls. Interestingly, the MIC of vancomycin (for the colonies recovered from the wound) was decreased after treatment with staphopain A in the animal models, too (Fig. 5).

Resistance to carbapenems variously changed after co-culture in the L-929 cell line and animal model. Doripenem as the most preferred antibiotic for the treatment of *P. aeruginosa* infections was inactivated by the microorganism in both in vitro and in vivo models. Whereas, the survival of *P. aeruginosa* was reduced in the co-culture model due to the synergistic effect of doripenem and staphopain A. The expression level of *kpc*, *mexAB-oprM*, and *oprD* changed in favor of doripenem susceptibility. Also, a synergistic relationship was detected between staphopain A and doripenem in the L-929 cell line. According to the data of the animal model, the MIC of doripenem was declined in treated groups comparing control groups (for the colonies recovered from the wound) (Fig. 5).

## Discussion

Biofilm formation plays a vital role in polymicrobial infection as a property to resist antimicrobial compounds, host’s immune system, and persist in competition strategy [18]. Therefore, anti-biofilm or biofilm-disrupting compounds could contribute to curing the polymicrobial infection. The current study focused on the effect of staphopain A- a staphylococcal cysteine protease- on dual-species biofilms in an animal model.

Enzymes secreted from bacteria play a double-edged sword, i.e., the secreted enzyme and physiological roles sometimes damage the adjacent bacteria. Nuclease hydrolyzes the eDNA and manipulates the biofilm of community-acquired *S. aureus* [19]. DNaseI also inhibits the cell aggregation of *P. aeruginosa* and *S. aureus* due to the role of eDNA as an intercellular adhesion [20, 21]. Moreover, previous studies reported that the biofilm disrupting property of staphylococcal cysteine proteases destroyed the established biofilm of *S. aureus* [13]. According to Fig. 1, two effects of staphopain A were observed. First, biofilm was significantly disrupted on days 1 to 5 after treatment, and the colony counts of *S. aureus* and *P. aeruginosa* increased 5–6 fold. That is suggested the noticeable biofilm-degrading effect of staphopain A on dual-species biofilm. Regarding the proteinous structure of mixed biofilm, the disrupting role of staphopain A is rational [22]. A similar effect was described for Dispersin B -a hexoaminidase secreted by *Aggregatibacter actinomycetemcomitans*, which hydrolyzes poly-(β-1,6)-n-acetylglucosamine (PNAG) of gram-positive bacteria, and inhibits the biofilm formation [23]. Second, staphopain A decreased the viability of *S. aureus* and *P. aeruginosa* during days 5 to 10. Enzymes are promising agents to overcome antibiotic-resistant and biofilm-forming bacteria. An extended-spectrum of enzymes including proteases, α-amylase, metalloproteases, and etc., kill bacteria through the degradation of vital macromolecules [24, 25].

As a communication system, QS translates and signals the cells to regulate biofilm formation, virulence, and resistance. Microorganisms are communicated by QS either cooperatively or competitively. In vitro models of polymicrobial infection suggested that QS leads to the competitive interspecies behavior. At the same time, other studies explained the cooperative relationship among different species. As depicted in Fig. 2, RNA II downregulated after treatment with staphopain A in both co- and mono-infection models. Likewise, *rhlI* and *lasI* expression levels in *P. aeruginosa* noticeably decreased following treatment. Despite the down-regulation of autoinducers, the response receptor– *rhlR* and *lasR* up-regulated compared to control groups. QS in *P. aeruginosa* is regulated positively or negatively in different ways, including vqsR, vfr, rmsA, etc. VqsR controls *lasI* transcription and AHL production through a positive feedback response [26].

Furthermore, *agr* expression level decreased as RNAII down-regulated. Although this alteration in gene expression level was detected in the abiotic and cell culture models as it was reported elsewhere [27, 28], RNAII up-regulated in the animal wound model, particularly in days 5 to 10 of co-infection. In contrast, in the staphopain treated group, the expression level of RNAII dramatically reduced, and virulence factor production decreased.

Biofilm formation plays a critical role in polymicrobial infections and contribution to antimicrobial resistance. Therefore, a biofilm-degrading compound might aid in curing the infection. Staphopain A degrades Staphylococcal biofilms [13]. In the current study, biofilm degradation through cysteine protease function was observed in dual-species. Staphopain A effectively degraded established biofilms and decreased the biofilm formation in the animal models, cell culture, and abiotic surfaces. DNase I and trypsin mixture contributed to biofilm dispersal in a wound-like media and decreased the minimum biofilm eradication concentration of meropenem and amikacin [22]. On the other hand, staphopain A treatment decreased the expression level of genes involved in *P. aeruginosa* and *S. aureus* biofilm formation. However, staphopain A has no regulatory effect on bacteria, it seems that it influenced some regulatory systems leading the biofilm formation.

In addition to QS and biofilm formation, alternative sigma factors’ expression level usually alters in dual-species interactions. The important sigma factor of *S. aureus*- *sigB* up-regulates in polymicrobial infections and critical regulators, especially in chronic biofilms [29, 30]. Although *sigB* and *agr* are regulated reciprocally and in an *ica*-independent manner (also observed in the control group), staphopain A effectively down-regulated all of them. *sigB* functions upstream of *agr* operon and regulates biofilm formation and appearance of small-colony variants (SCV) [29]. Based on the phenotypic investigations, some tiny, slow-growing *S. aureus* colonies were detected in the co-culture with *P. aeruginosa*, which were hemolysis negative; however, these colonies were not confirmed as SCVs. The mentioned phenotypes were not observed after treatment with staphopain A, whether in a murine model of wound or cell culture. Furthermore, *sigB* up-regulated slightly in the so-called isolates in murine wound infection and L-929 models. The acute wound model suggested that *sigB* expression is not as crucial as in the chronic models, as similar findings were reported on the ability of *sigB* mutants to co-colonize with *P. aeruginosa*. The authors concluded that *sigB* might be more critical in chronic infections rather than acute ones [31].

The interaction of *S. aureus* and *P. aeruginosa* leads to alteration in virulence and antibiotic resistance. As depicted in Fig. 4, the production of pyocyanin and Las proteases (LasA and LasB) decreased significantly following treatment with staphopain A in both mono- and co-infection groups. The mentioned virulence factors are QS-regulated, and down-regulation of QS systems led to a reduction in virulence factor production [14]. Furthermore, antibiotic susceptibility changed after staphopain A treatment.

In the current study, *S. aureus* isolates extensively resisted vancomycin following the co-culture with *P. aeruginosa*. Moreover, resistance to doripenem increased after co-culture with *S. aureus*. Various studies described the stimulatory effect of *P. aeruginosa* exoproducts, including 2-heptyl-4-hydroxyquinoline-N-oxide (HQNO) and LasA, on antibiotic resistance in *S. aureus*. For illustration, HQNO draws *S. aureus* forward tobramycin resistance [32]. Likewise, alginate produced by *P. aeruginosa* leads to reduced susceptibility to vancomycin [8]. *S. aureus* exoproducts such as peptidoglycan particles cause resistance to tobramycin and aminoglycosides in *P. aeruginosa* [33]. Interestingly, staphopain A treatment caused a reduction in resistance to doripenem, which was mediated by a change in the expression level of *kpc*, *mexAM-oprM*, and *oprD* in favor of susceptibility. Although *P. aeruginosa* isolate was resistant to carbapenems, staphopain A treatment contributed to carbapenemase down-regulation. Strikingly, *S. aureus* isolate used in this study, was susceptible to vancomycin. When the isolate was co-cultured with *P. aeruginosa*, it became vancomycin-resistant (MIC: 128 µg/mL). The expression level of *walk/R* system altered, which resulted in the production of thick peptidoglycan and resistance to vancomycin [34]. Regarding the alteration of vancomycin susceptibility, it is suggested that staphopain A resulted in suppression of *P. aeruginosa* exoproducts mediated resistance to this antibiotic. However, the exact mechanism is not clear.

Concludingly, staphopain A might be a promising compound to combine with antibiotics to cure polymicrobial infections. However, many questions remain to answer about it. Many polymicrobial infections are strain-dependent, and the efficacy of staphopain A on biofilm in such infection should be evaluated.

### Electronic supplementary material

Below is the link to the electronic supplementary material.

Supplementary Material 1

Supplementary Material 2

## Data Availability

The data can be accessible to the interested researchers by the corresponding authors on reasonable request.

## References

[1] Ahmed EF, Rasmi AH, Darwish AMA, Gad GFM (2023). Prevalence and resistance profile of bacteria isolated from wound infections among a group of patients in upper Egypt: a descriptive cross-sectional study. BMC Res Notes.

[2] Kim H-J, Na SW, Alodaini HA, Al-Dosary MA, Nandhakumari P, Dyona L (2021). Prevalence of multidrug-resistant bacteria associated with polymicrobial infections. J Infect Public Health.

[3] Sandmann S, Nunes JV, Grobusch MP, Sesay M, Kriegel MA, Varghese J, Schaumburg F (2023). Network analysis of polymicrobial chronic wound infections in Masanga, Sierra Leone. BMC Infect Dis.

[4] Tahmasebi H, Dehbashi S, Arabestani MR (2021). Antibiotic resistance alters through iron-regulating Sigma factors during the interaction of Staphylococcus aureus and Pseudomonas aeruginosa. Sci Rep.

[5] Tahmasebi H, Dehbashi S, Jahantigh M, Arabestani MR (2020). Relationship between biofilm gene expression with antimicrobial resistance pattern and clinical specimen type based on sequence types (STs) of methicillin-resistant S. Aureus. Mol Biol Rep.

[6] Boles BR, Horswill AR (2008). Agr-mediated dispersal of Staphylococcus aureus biofilms. PLoS Pathog.

[7] Lauderdale KJ, Malone CL, Boles BR, Morcuende J, Horswill AR (2010). Biofilm dispersal of community-associated methicillin-resistant Staphylococcus aureus on orthopedic implant material. J Orthop Research: Official Publication Orthop Res Soc.

[8] Orazi G, O’Toole GA (2017). <em > Pseudomonas aeruginosa alters < em > Staphylococcus aureus Sensitivity to Vancomycin in a Biofilm model of cystic fibrosis Infection</em >. mBio.

[9] Biswas R, Martinez RE, Gohring N, Schlag M, Josten M, Xia G (2012). Proton-binding capacity of Staphylococcus aureus wall teichoic acid and its role in controlling autolysin activity. PLoS ONE.

[10] Kali A, Bhuvaneshwar D, Charles PM, Seetha KS (2016). Antibacterial synergy of curcumin with antibiotics against biofilm producing clinical bacterial isolates. J Basic Clin Pharm.

[11] Kantyka T, Plaza K, Koziel J, Florczyk D, Stennicke HR, Thogersen IB (2011). Inhibition of Staphylococcus aureus cysteine proteases by human serpin potentially limits staphylococcal virulence. Biol Chem.

[12] Sonesson A, Przybyszewska K, Eriksson S, Mörgelin M, Kjellström S, Davies J (2017). Identification of bacterial biofilm and the Staphylococcus aureus derived protease, staphopain, on the skin surface of patients with atopic dermatitis. Sci Rep.

[13] Mootz JM, Malone CL, Shaw LN, Horswill AR (2013). Staphopains Modulate < span class=named-content genus-species id=named-content-1>Staphylococcus aureus Biofilm Integrity. Infect Immun.

[14] Limoli DH, Warren EA, Yarrington KD, Donegan NP, Cheung AL, O’Toole GA (2019). Interspecies interactions induce exploratory motility in Pseudomonas aeruginosa. eLife.

[15] Filkins LM, Graber JA, Olson DG, Dolben EL, Lynd LR, Bhuju S, O’Toole GA (2015). Coculture of Staphylococcus aureus with Pseudomonas aeruginosa drives S. Aureus towards Fermentative Metabolism and reduced viability in a cystic fibrosis model. J Bacteriol.

[16] Grada A, Mervis J, Falanga V (2018). Research Techniques made simple: animal models of Wound Healing. J Invest Dermatology.

[17] Dehbashi S, Alikhani MY, Tahmasebi H, Arabestani MR (2021). The inhibitory effects of Staphylococcus aureus on the antibiotic susceptibility and virulence factors of Pseudomonas aeruginosa: A549 cell line model. AMB Express.

[18] Zahedani SS, Tahmasebi H, Jahantigh M (2021). Coexistence of virulence factors and efflux pump genes in clinical isolates of *Pseudomonas aeruginosa*: analysis of Biofilm-forming strains from Iran. Int J Microbiol.

[19] Kiedrowski MR, Kavanaugh JS, Malone CL, Mootz JM, Voyich JM, Smeltzer MS (2011). Nuclease modulates biofilm formation in community-associated methicillin-resistant Staphylococcus aureus. PLoS ONE.

[20] Tetz GV, Artemenko NK, Tetz VV (2009). Effect of DNase and antibiotics on biofilm characteristics. Antimicrob Agents Chemother.

[21] Allesen-Holm M, Barken KB, Yang L, Klausen M, Webb JS, Kjelleberg S (2006). A characterization of DNA release in Pseudomonas aeruginosa cultures and biofilms. Mol Microbiol.

[22] Fanaei Pirlar R, Emaneini M, Beigverdi R, Banar M, van Leeuwen B, Jabalameli W (2020). Combinatorial effects of antibiotics and enzymes against dual-species Staphylococcus aureus and Pseudomonas aeruginosa biofilms in the wound-like medium. PLoS ONE.

[23] Kropec A, Maira-Litran T, Jefferson KK, Grout M, Cramton SE, Götz F (2005). Poly-N-acetylglucosamine production in Staphylococcus aureus is essential for virulence in murine models of systemic infection. Infect Immun.

[24] Vachher M, Sen A, Kapila R, Nigam A (2021). Microbial therapeutic enzymes: a promising area of biopharmaceuticals. Curr Res Biotechnol.

[25] Lehouritis P, Springer C, Tangney M (2013). Bacterial-directed enzyme prodrug therapy. J Controlled Release.

[26] Juhas M, Wiehlmann L, Huber B, Jordan D, Lauber J, Salunkhe P (2004). Global regulation of quorum sensing and virulence by VqsR in Pseudomonas aeruginosa. Microbiology.

[27] Woods PW, Haynes ZM, Mina EG, Marques CNH. Maintenance of S. Aureus in co-culture with P. Aeruginosa while growing as Biofilms. Front Microbiol. 2019;9(3291). 10.3389/fmicb.2018.03291 PMC6333908 30687276

[28] Goerke C, Wolz C (2004). Regulatory and genomic plasticity of Staphylococcus aureus during persistent colonization and infection. Int J Med Microbiol.

[29] Mitchell G, Séguin DL, Asselin A-E, Déziel E, Cantin AM, Frost EH (2010). Staphylococcus aureus Sigma B-dependent emergence of small-colony variants and biofilm production following exposure to Pseudomonas aeruginosa 4-hydroxy-2-heptylquinoline-N- oxide. BMC Microbiol.

[30] Mitchell G, Fugère A, Pépin Gaudreau K, Brouillette E, Frost EH, Cantin AM, Malouin F (2013). SigB is a Dominant Regulator of Virulence in Staphylococcus aureus small-colony variants. PLoS ONE.

[31] Millette G, Langlois J-P, Brouillette E, Frost EH, Cantin AM, Malouin F. Despite antagonism in vitro, Pseudomonas aeruginosa enhances Staphylococcus aureus colonization in a murine lung infection model. Front Microbiol. 2019;10(2880). 10.3389/fmicb.2019.02880 PMC6923662 31921058

[32] Radlinski L, Rowe SE, Kartchner LB, Maile R, Cairns BA, Vitko NP (2017). Pseudomonas aeruginosa exoproducts determine antibiotic efficacy against Staphylococcus aureus. PLoS Biol.

[33] Trizna EY, Yarullina MN, Baidamshina DR, Mironova AV, Akhatova FS, Rozhina EV (2020). Bidirectional alterations in antibiotics susceptibility in Staphylococcus aureus—Pseudomonas aeruginosa dual-species biofilm. Sci Rep.

[34] Hu Q, Peng H, Rao X. Molecular Events for Promotion of Vancomycin Resistance in Vancomycin Intermediate Staphylococcus aureus. Front Microbiol. 2016;7(1601). 10.3389/fmicb.2016.01601 PMC5062060 27790199

