# Supplementary material for: Staphopain mediated virulence and antibiotic resistance alteration in co-infection of Staphylococcus aureus and Pseudomonas aeruginosa: an animal model
Source: BMC Biotechnol. 2024 Mar 4;24:10. doi: 10.1186/s12896-024-00840-x (PMC10913572; doi:10.1186/s12896-024-00840-x)
Supplement: Supplementary file 1 — Supplementary Material 1 [file 12896_2024_840_MOESM1_ESM.docx]

**Supplementary File**

**The cloning and expression of *scpA* gene:**

1. Primer design: The forward and reverse primers were designed in SnapGene software (GSL Biotech LLC). The sequence of *scpA* in *Staphylococcus aureus* was selected from gene bank. Regarding the cutting sites of the restriction enzymes and the sequence of the gene, *BamHI* and *XhoI* were selected. The restriction sequences were added to the primers and the final sequences of primers were designed as follows:

| Gene | Sequence | Tm |
| --- | --- | --- |
| *scpA* | F: GGG GGA TCC ATG AGCAATTCAAATATCAAAGC  R: GGG GGG GAG CTC TT AAT AAC CAT AAA TAG ATG | 59ºC |

1. Gene amplification: The gene was amplified using PCR method. 25 µL of the final solution containing 1 µL DNA templates, 0.5 µL of each primer with a concentration of 25 pM and 12.5 µL of PCR Master Mix (Ampliqon, Denmark) was used for PCR reaction. The PCR product was purified from agarose gel using Qiagen purification kit according to manufacturer’s instruction.
2. Double digestion: The insert and vector were double-digested by *BamHI* and *XhoI* as follows: 20 µL of vector and purified PCR product, 21 µL nuclease free distilled water, 1 µL BamHI, 3 µL XhoI, 5 µL of H buffer, briefly mixed with a sterile tip. Then, the mixture was incubated at 37 ºC for 2 hours. Subsequently, inactivated at 60 ºC for 5 minutes. The digestion’s product was gel-purified using Qiagen purification kit.
3. Ligation: The vector and insert were mixed in a 1:5 proportion and 1.5 µL buffer and 0.5 µL T_4_ ligase (Thermofisher Scientific, USA). The mixture was incubated at 4 ºC for 10 minutes, then the temperature increased to 22 ºC for 1 hour.
4. Transformation: *E. coli* TOP10 was selected to prepare competent cells using CaCl_2_ method. 5 mL of *E. coli* TOP10 in LB broth (exponential phase) were centrifuged at 9000 rpm for 3 min. Then, 200 µL of cold CaCl_2_ was added to the precipitate and incubated on ice for 30 min. The centrifugation and incubation steps were repeated again. Afterwards, 100 µL of CaCl_2_ was added to the precipitate and stored at 4ºC.

10 µL of ligated vector was added to 100 µL of competent cells and incubated on ice for 20 min. Then, the suspension was immediately incubated at 42 ºC for 90 seconds. One mL of fresh LB broth was added to the suspension and incubated at 37 ºC for 1h. Subsequently, the mixture was plated on LB agar supplemented with kanamycin (50 µg/mL) with sterile beads and incubated at 37 ºC overnight.

1. Confirmation of recombinant vector: 3-5 colonies grown on LB agar were selected for colony PCR. The recombinant vector was confirmed using T7 and *scpA* primers. Also, the vector was extracted by GeneAll plasmid purification kit (GeneAll, South Korea) and sequenced to confirm the nucleotide sequence (Pishgam, Iran).
2. Expression and overexpression: *E. coli*BL21 was chosen as expression host and prepared as competent cells as it was described earlier. 2 µL of recombinant vector was transformed to the competent cells and then plated on LB agar supplemented with kanamycin (50 µg/mL) as mentioned above.

One colony was suspended in 5 mL LB broth supplemented with kanamycin and incubated at 37 ºC overnight. 5 mL of *E. coli*BL21 in exponential phase (OD_600_: 0.6) was prepared for expression, then, 50 µL IPTG (Isopropyl β- d-1-thiogalactopyranoside, 100 mM) was added to culture and incubated at 37 ºC shaken at 100 rpm. . 1 mL of the culture was transferred to a new sterile microtube before addition of IPTG and 2 and 4 hours after induction. The expression of recombinant protein was investigated using SDS-PAGE and confirmed by anti his-tagged western blotting technic.

1. Protein purification: The construct protein was purified using cobalt column chromatography as described in Afshar et.al (1) and protein purification manual of pET systems. Briefly, 300 mL of LB broth supplemented with kanamycin was inoculated by 5 mL of overnight culture of recombinant *E. coli*BL21 and incubated at 37 °C for 2 hours until reached OD_600_:0.6. Then, 180 µL of IPTG was added and the media was incubated at 20 °C for 16 hours. Subsequently, the bacteria precipitated at 5000 rpm for 5 min. The precipitant was suspended in sucrose buffer and incubated on ice and EDTA was added gently. After incubation, the suspension was centrifuged at 8500 rpm for 20 min and MgSO_4_ was added to the precipitant. Then, the suspension was shaken in water bath for 10 min, after centrifugation the supernatant was dialyzed against lysis buffer, mixed with cobalt resin, incubated at 4 °C in shaker incubator for 60 min, and added to the column. After drainage, the resin was washed twice with wash buffer. Finally, the protein was eluted with elution buffer.

**Virulence factor production**

Pyocyanin, biofilm, LasA protease, and motility (swarming and swimming) were investigated on the bacteria recovered from the planktonic and biofilm states of co-culture.

Briefly, to investigate the LasA activity of *P. aeruginosa* strains, an overnight culture of *S. aureus* ATCC25923 was boiled for 10 minutes, precipitated by centrifugation, and the pellet was resuspended in PBS to OD_595_:0.8. Then, the overnight broth cultures of *P. aeruginosa* strains were precipitated at 10000 rpm for 10 minutes resuspended in 100 µl of CDMC solution. The OD_595_ was read in 5-minute intervals. To measure pyocyanin production in pseudomonas strains, the chloroform and HCl method was used based on Moayedi and et. al. (2). Then, the extracted pyocyanin was measured spectrophotometrically in OD_520_ and was multiplied by 17.027. To investigate biofilm formation, the crystal violet method modified by O'Toole was employed (3). Based on the studies of Deziel *et al.* (4), and Murray *et al.* (5), the swarming and swimming motilities were investigated using nutrient broth supplemented with 0.5 % and 0.3% agar, respectively.

1. Afshar D, Pourmand MR, Jeddi-Tehrani M, Yaraghi AAS, Azarsa M, Shokri F. Fibrinogen and fibronectin binding activity and immunogenic nature of choline binding protein M. Iranian journal of public health. 2016;45(12):1610.

2. Moayedi A, Nowroozi J, Sepahy AA. Effect of fetal and adult bovine serum on pyocyanin production in Pseudomonas aeruginosa isolated from clinical and soil samples. Iranian journal of basic medical sciences. 2017;20(12):1331-8.

3. O'Toole GA. Microtiter dish biofilm formation assay. J Vis Exp. 2011(47).

4. Deziel E, Lepine F, Milot S, Villemur R. rhlA is required for the production of a novel biosurfactant promoting swarming motility in Pseudomonas aeruginosa: 3-(3-hydroxyalkanoyloxy)alkanoic acids (HAAs), the precursors of rhamnolipids. Microbiology (Reading, England). 2003;149(Pt 8):2005-13.

5. Murray TS, Ledizet M, Kazmierczak BI. Swarming motility, secretion of type 3 effectors and biofilm formation phenotypes exhibited within a large cohort of Pseudomonas aeruginosa clinical isolates. Journal of medical microbiology. 2010;59(Pt 5):511-20.

**Results**

1. As depicted in Figure 1, the recombinant vector was confirmed. The 1180 bp fragment was detected on 1% agarose gel. Also, the purified recombinant vector is shown in Figure 1.


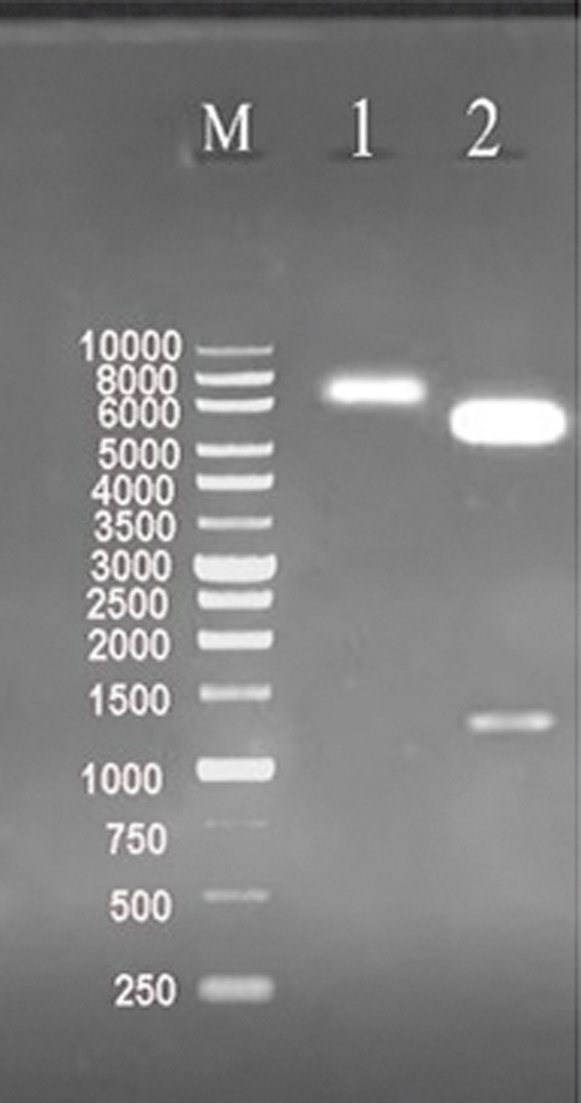


SF1. The gel electrophoresis of recombinant vector. M: 1kb marker, Lane 1: The 6416bp recombinant vector, Lane 2: The double-digested recombinant vector including the 5200bp vector band and 1180bp insert fragment.

1. According to Figure 2, the recombinant protein was expressed successfully in the expression host and detected on SDS-PAGE. The 44.2 KDa recombinant protein was purified and the PI was determined as 9.64. The recombinant protein was confirmed by anti His-tag western blotting shown in Figure 3.


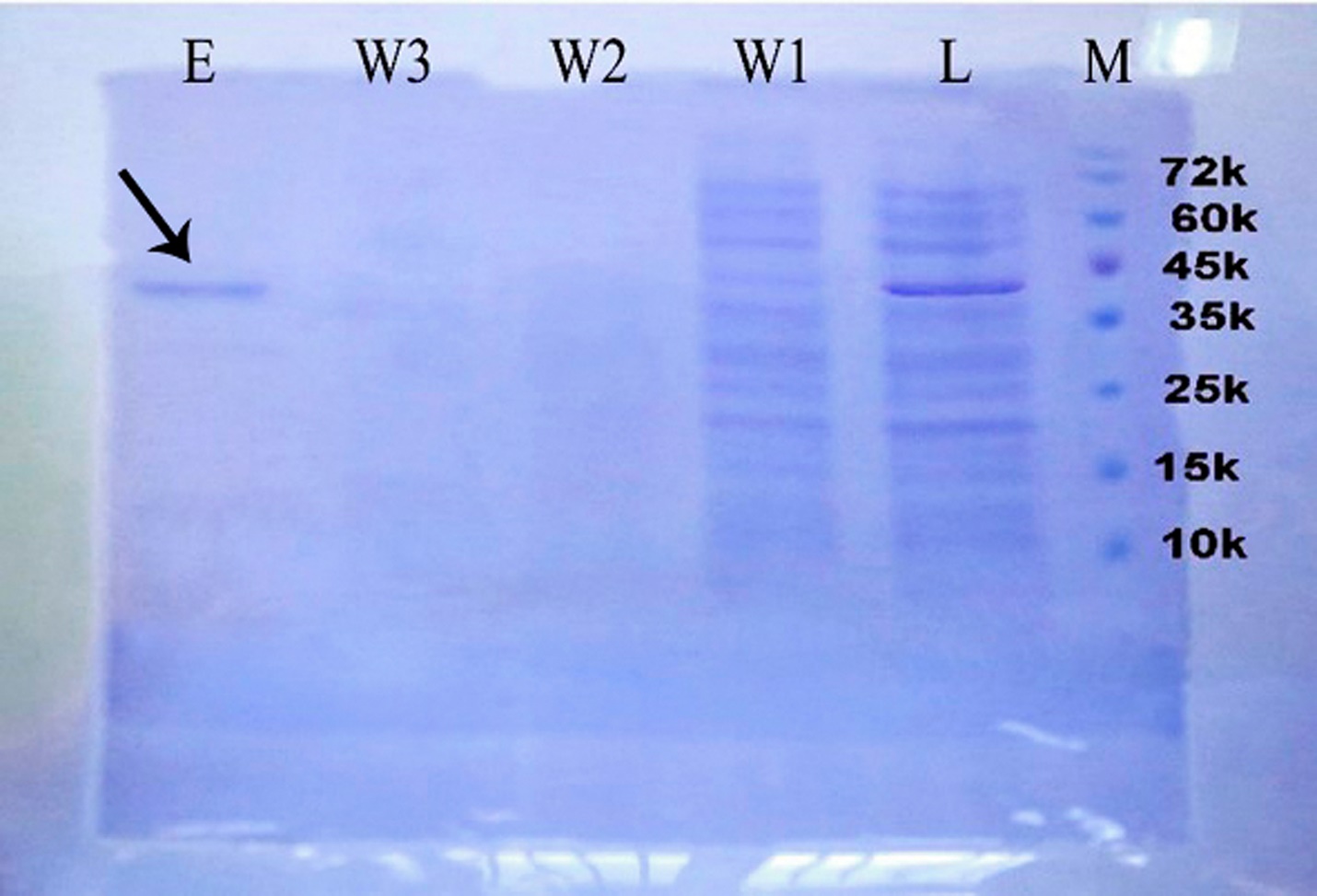


SF2. The SDS-PAGE of purified protein. M: All Blue Regular Range Protein Marker (10- 180 kDa). Lane L: The lysate of expression host in LB broth. The recombinant protein is shown in 44.2 kDa. Lane W_1_-W_3_: The washing stages of protein purification. Lane E: The recombinant protein eluted and is indicated at 44.2 kDa by a black arrow.


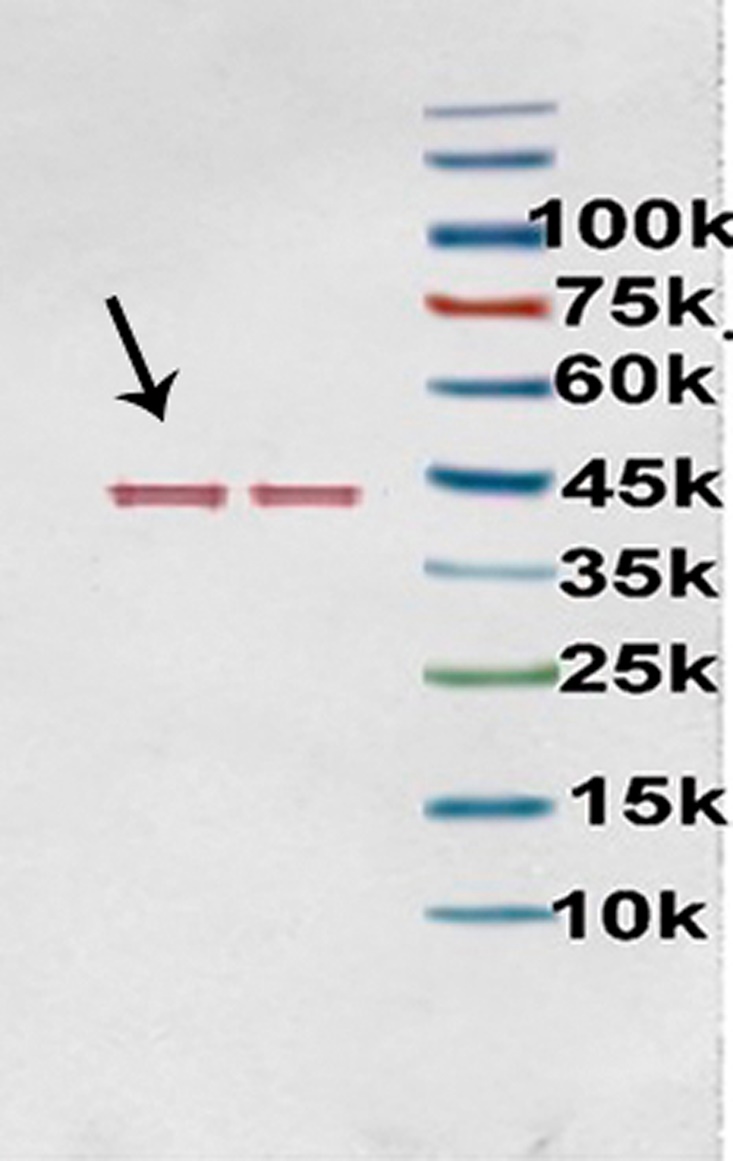


SF3. The anti His-tagged western blotting of recombinant protein. M: The prestained protein marker (10-180 kDa). Lane 1 and 2: The recombinant protein attached to antibody and stained with DAB, indicated by black arrow.

1. The concentration and functionality of enzyme was determined as 15mg/mL and 1730 Unit/mL.
