# Supplementary material for: Staphopain mediated virulence and antibiotic resistance alteration in co-infection of Staphylococcus aureus and Pseudomonas aeruginosa: an animal model
Source: BMC Biotechnol. 2024 Mar 4;24:10. doi: 10.1186/s12896-024-00840-x (PMC10913572; doi:10.1186/s12896-024-00840-x)
Supplement: Supplementary file 2 — Supplementary Material 2 [file 12896_2024_840_MOESM2_ESM.docx]

**Supplementary File**

**Cytotoxicity Test**

The cytotoxicity of the enzyme was assess at 256 µg/mL more than 30 percent.

SF4. The cytotoxicity of Staphopain A on L-929 cell line

**Synergistic effect between Staphopain A and Vancomycin**

64 µg/mL Staphopain A were combined to the concentrations of 256 µg/mL, 64 µg/mL, 16 µg/mL, and 4 µg/mL of vancomycin and then inoculated to the co- and mono-cultures of *S. aureus* and *P. aeruginosa* on L-929 cell line. The co- and mono-cultures on L-929 were prepared according to Co-culture on fibroblast cell line section described in the main manuscript.

After 48 h, a remarkable synergistic effect of vancomycin and Staphopain A was observed. The viability and frequency of recovered isolates reduced notably (p < 0.0001). The biofilm assessment based on the crystal violet assay and the trypan blue assay for viable cell count indicated the enzyme disrupted the biofilm and vancomycin killed *S.aureus*, however; this activity was not detected in *P.aeruginosa*.


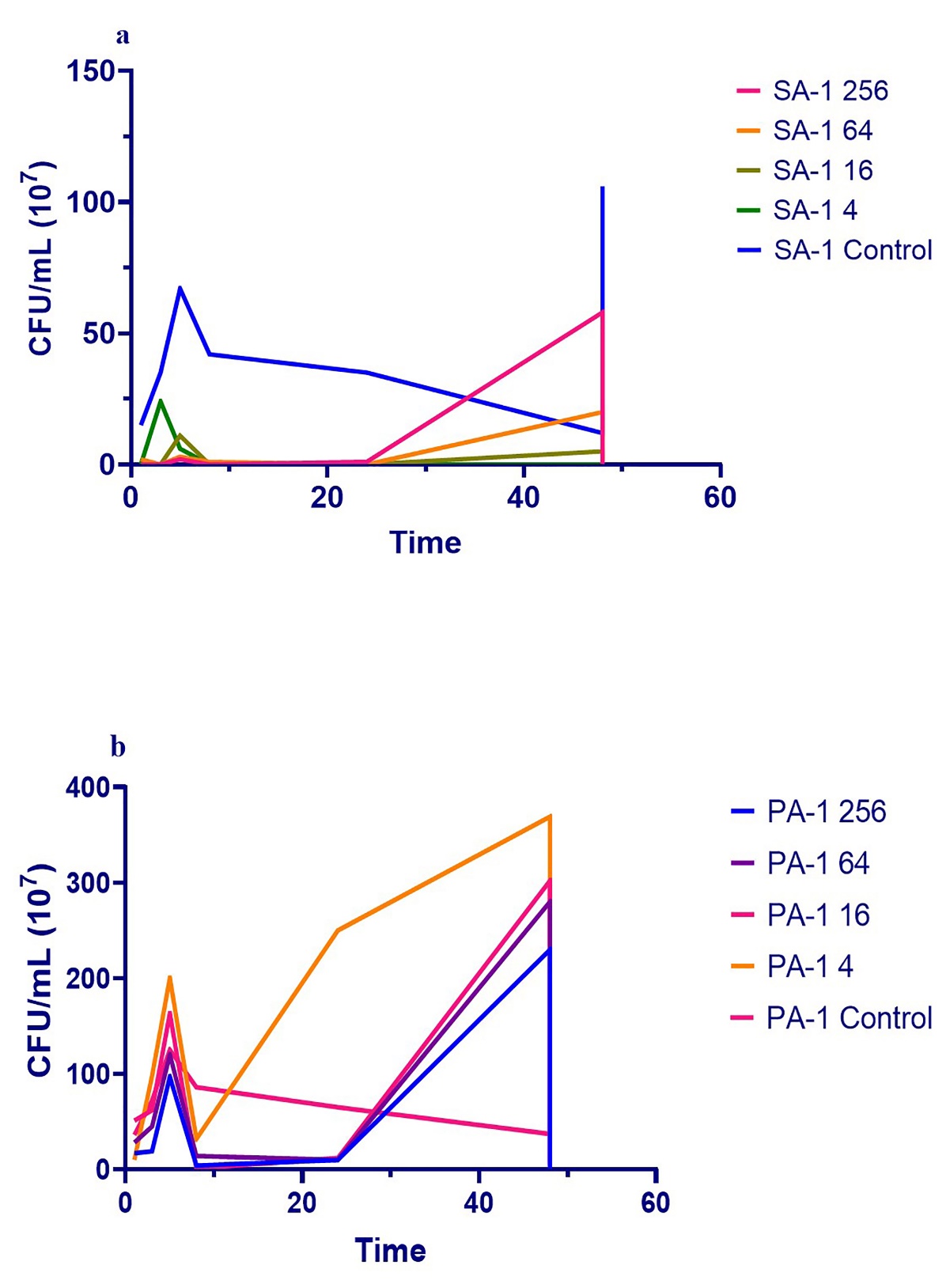


SF5. Synergistic effect between Staphopian A and Vancomycin. A: The synergistic effect on S.aureus isolates. The Isolates recovered from biofilm and the viability after 48 h treatment with the combination of Staphopian A and vancomycin. B: The synergistic effect on P.aeruginosa isolates. The Isolates recovered from biofilm and the viability after 48 h treatment with the combination of Staphopian A and vancomycin
